# Taxonomic and Numerical Resolutions of Nepomorpha (Insecta: Heteroptera) in Cerrado Streams

**DOI:** 10.1371/journal.pone.0103623

**Published:** 2014-08-01

**Authors:** Nubia França da Silva Giehl, Karina Dias-Silva, Leandro Juen, Joana Darc Batista, Helena Soares Ramos Cabette

**Affiliations:** 1 Programa de Pós-graduação em Ecologia e Conservação, Universidade do Estado de Mato Grosso (UNEMAT), Nova Xavantina, Mato Grosso, Brasil; 2 Programa de Pós-graduação em Ciências Ambientais, ICB1, Universidade Federal de Goiás, Goiânia, Goiás, Brasil; 3 Laboratório de Ecologia e Conservação, Instituto de Ciências Biológicas, Universidade Federal do Pará (UFPA), Belém, Pará, Brasil; 4 Laboratório de Entomologia, Universidade do Estado de Mato Grosso (UNEMAT), Nova Xavantina, Mato Grosso, Brasil; 5 Departamento de Biologia, Universidade do Estado de Mato Grosso (UNEMAT), Nova Xavantina, Mato Grosso, Brasil; Universidade de São Paulo, Faculdade de Filosofia Ciências e Letras de Ribeirão Preto, Brazil

## Abstract

Transformations of natural landscapes and their biodiversity have become increasingly dramatic and intense, creating a demand for rapid and inexpensive methods to assess and monitor ecosystems, especially the most vulnerable ones, such as aquatic systems. The speed with which surveys can collect, identify, and describe ecological patterns is much slower than that of the loss of biodiversity. Thus, there is a tendency for higher-level taxonomic identification to be used, a practice that is justified by factors such as the cost-benefit ratio, and the lack of taxonomists and reliable information on species distributions and diversity. However, most of these studies do not evaluate the degree of representativeness obtained by different taxonomic resolutions. Given this demand, the present study aims to investigate the congruence between species-level and genus-level data for the infraorder Nepomorpha, based on taxonomic and numerical resolutions. We collected specimens of aquatic Nepomorpha from five streams of first to fourth order of magnitude in the Pindaíba River Basin in the Cerrado of the state of Mato Grosso, Brazil, totaling 20 sites. A principal coordinates analysis (PCoA) applied to the data indicated that species-level and genus-level abundances were relatively similar (>80% similarity), although this similarity was reduced when compared with the presence/absence of genera (R = 0.77). The presence/absence ordinations of species and genera were similar to those recorded for their abundances (R = 0.95 and R = 0.74, respectively). The results indicate that analyses at the genus level may be used instead of species, given a loss of information of 11 to 19%, although congruence is higher when using abundance data instead of presence/absence. This analysis confirms that the use of the genus level data is a safe shortcut for environmental monitoring studies, although this approach must be treated with caution when the objectives include conservation actions, and faunal complementarity and/or inventories.

## Introduction

Human activities generate serious impacts on natural aquatic environments, especially by replacing the vegetation with plantations and pastures [1], constructing dams and reservoirs, and diverting the natural course of waterways [2], [3]. These processes, either alone or in synergy, result in decreased habitat heterogeneity, increased input of sediments into the channel, and the loss of aquatic biodiversity [4], [5].

Due to major environmental problems and limited resources for evaluating diversity, researchers have resorted to relatively fast and inexpensive methods to evaluate and monitor aquatic ecosystems [6]–[9]. In particular, while invertebrates are widely used for the monitoring of freshwater systems, they are prone to a “Linnean deficit” [10], in which closely-related species have been diagnosed based on subtle or even subjective morphological characters, and many have still not been described [11]. The lack of appropriate identification keys may also contribute to the lack of an accurate taxonomic resolution in many studies, few of which are capable of working at the species level. More often than not, then, higher taxonomic levels have been analyzed, and this has been justified based on arguments such as an effective cost-benefit ratio, the time needed for processing samples [8], limited resources [12], a lack of taxonomists and/or data on the ranges and ecological requirements of the species [11], [13].

Given the difficulties and demands for rapid methods for bioevaluation, a priority issue is understanding the level of taxonomic identification required for studies of anthropogenic impacts and the monitoring of aquatic systems [14]. The question of numerical resolution–whether abundance or presence/absence data are more effective for the analysis of patterns of biodiversity and environmental quality–has also been evaluated [9], [15]. The use of numerical resolution may be an alternative that reduces sample processing time and allows a comparison of results using different methods of inventory and monitoring, and across different regions [16].

Insects of the order Heteroptera (Nepomorpha and Gerromorpha) have aquatic and semi-aquatic habits, are widely distributed, and vary considerably in form and niche, allowing them to occupy a diversity of habitats, including both lentic and lotic bodies of water [17]. Most heteropterans live exclusively in water from the nymphal stage until becoming adults, as in the case of the Nepomorpha. These organisms are ecologically-important predators, and provide a variety of ecological services, including: (i) the biological control of disease vectors, such as the larvae of *Anopheles*, *Culex*, and *Aedes* mosquitoes [18], [19] and *Biomphalaria* snails [20]; (ii) the predation of aquatic and terrestrial organisms at the water/air interface; (iii) serving as prey for fish, amphibians, and birds [21]; and (iv) being able to respond to environmental disturbances [17], [22], [23].

Nepomorpha have only recently become the subjects of environmental evaluation or conservation studies [e.g. 22–26]. In brasilian studies, difficulties have been encountered with regard to the identification of species, which are exacerbated by the lack of reference material, which is often dispersed and inaccessible. On the other hand, identification at the generic level is relatively simple, being based on well-defined characters. The time necessary for the identification of genera tends to be much shorter, the results more reliable, and there is no need to submit the material to experts.

Based on these premises, this study aims to investigate the level of congruence in the data matrices of Nepomorpha genera versus species, by applying taxonomic and numerical resolutions and analyzing whether congruence remains similar when analyzing altered and preserved sites separately, in order to answer to the following questions: a) how much information is lost when the analyses are conducted with a genus-level data matrix in comparison with a species-level one; and b) whether the ordinating patterns obtained from the incidence (presence/absence) of genera and/or species are similar to those obtained from abundance data. A marked congruence of genus-level data with those for species is assumed to support the use of the former for environmental-monitoring studies, enabling quicker decision-making by managers of affected populations, and scholars, when dealing with areas with high habitat-conversion rates.

## Materials and Methods

The last author, HSRC, has a permanent license to collect scientific specimens of aquatic insects (14457-1) granted by IBAMA/SISBIO, an organ of the Brazilian Environment Ministry, according to federal legislation. The areas where the specimens were collected are privately owned (Appendix S1), but prior permission was obtained from the owners or managers. None of the specimens collected in work represented IACUC-registered or endangered species.

### Study area

The Pindaíba River Basin is a right-bank tributary of the middle Mortes River, located in the southwestern state of Mato Grosso, Brazil, and its basin includes the municipalities of Barra do Garças, Araguaiana, Cocalinho, and Nova Xavantina. The region’s climate is Aw in the Köppen classification, with two clearly distinct seasons: dry and rainy [27]. Mean annual rainfall ranges from 1500 mm to 1800 mm, and temperatures from 18.9°C to 33.7°C [28].

**Figure 1:**
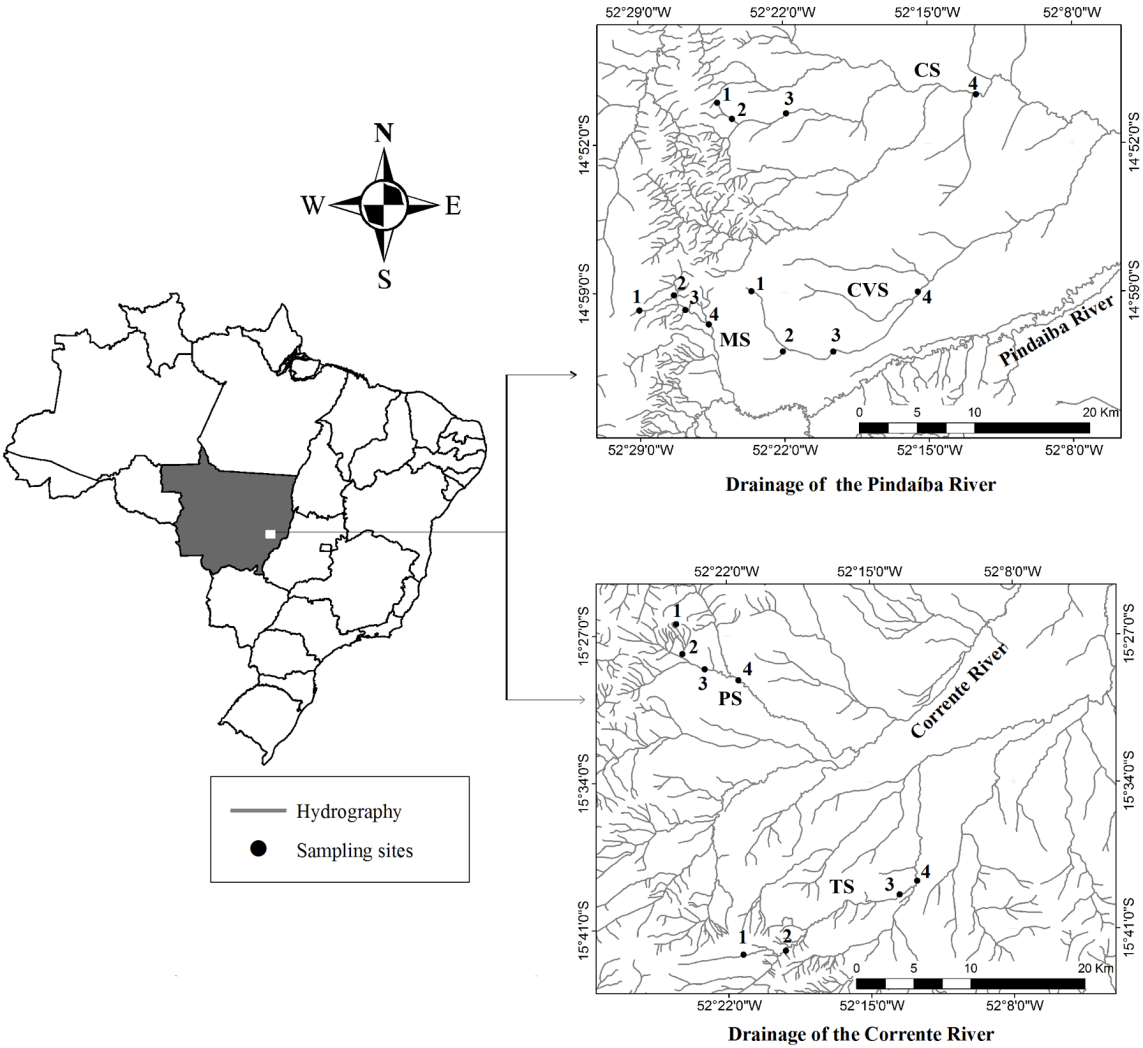
Sampling sites in the drainage basins of the Pindaíba and Corrente rivers, Mato Grosso, Brazil. (CS–Cachoeirinha Stream; CVS–Caveira Stream; MS–Mata Stream; PS–Papagaio Stream; TS–Taquaral Stream; 1^st^ to 4^th^–stream orders).

The study was carried out in stream sections ranging from the first to the fourth order (classification proposed by [29]). In this classification, first-order watercourses have no tributaries, and when two first order tributaries are connected, a second-order stream is formed. Thus two combined *n* order streams form a river of (n+1) order. A total of 20 sampling sites (Figure 1), with different conservation status were sampled in the Cachoeirinha, Caveira, Da Mata, Papagaio and Taquaral streams. The sites were visually evaluated using the Habitat Integrity Index (HII) [30]. This index, adapted from Petersen’s protocol [31] for small brazilian streams, is composed of twelve questions that describe the pattern of land use outside the riparian forest, riparian forest conditions, and stream channel characteristics. The HII provides values from 0 to 1, and the closer the value is to 1, the more pristine is the stream.

Environments with a HII of less than 0.70 presented considerable deforestation on one bank and the riparian vegetation, when it existed, was no more than a narrow strip. Land use outside the riparian vegetation consisted of pastures, with periodic access of cattle to the streams, resulting in banks with excavations or deep rutting. The most disturbed stream had a weir just upstream from the section sampled. Environments with HII≥0.70 varied from areas with clearly defined riparian vegetation (from 5 to 30 m wide) to environments with intact forest without gaps in their canopy. Sites with indices closer to 0.70 had extensive pasture or soybean plantations outside the riparian vegetation.

### Collection and identification

The specimens of Heteroptera (Nepomorpha) were collected in the rainy (January), dry (June/July), and early rainy (October/November) seasons in 2005, except in the Caveira Stream, which was sampled in 2008 during the same periods of the year. For sampling, we demarcated a 100 m transect in each of the streams, subdivided into twenty 5-meter segments. In each segment, a 18 cm sieve with a 250 µm mesh were applied three times, constituting a sample with 20 sites per stream/order in each season [24].

Specimens were preserved in 85% ethyl alcohol and identified by means of dichotomous keys [32]–[37]. Whenever necessary, specimens were analyzed by experts from the Federal University of the Pampa (UNIPAMPA) and the Federal University of Minas Gerais (UFMG). Samples were deposited in the “James Alexander Ratter” Zoo-Botanical Collection at the Nova Xavantina *campus* of Mato Grosso State University (UNEMAT), Mato Grosso, Brazil.

### Data analysis

In order to minimize data-normality problems, we used the logarithmic transformation log (x+1) in the abundance matrices. To test whether the data matrices were congruent, the analyses were initially conducted including all sites (r_all_, 20 sites with HII between 0.51 and 0.96) and, then, in order to determine whether congruence was sustained, we repeated the analyses separating the environments classified as “altered” (r_alter_; 10 sites, HII<0.70) from those identified as “preserved” (r_preserv_; 10 sites, HII≥0.70).

A Principal Coordinates Analysis (PCoA) was used to order sampling sites. The Bray-Curtis distance matrix was used for the abundance data and the Jaccard similarity matrix for presence/absences data (incidence), generating eigenvectors which were subsequently used in the Procrustes analysis [38]. This method was used to evaluate the degree of congruence of the Nepomorpha community, comparing the abundance of species and genera (taxonomic resolution), species abundance *versus* the presence/absence of species and genera, and finally, the abundance of genera *versus* their incidence (numerical resolution).

This is an overlapping method that compares each ordination pair using a rotational algorithm (rotational-fit), which finds the best fit between the corresponding geometric ordinations and, provides a correlation value, r (the square root of 1−m^2^). Values closer to 0 indicate a greater difference between ordination patterns and a value of 1 indicates complete overlap between the matrices [38], [39]. The fits were quantified and tested for statistical significance (p<0.05) using the Monte Carlo test (with 10,000 permutations). By using all the PCoA axes, we sought to represent the total variance of the system and enable the visualization of all the dimensions of the composition of the community. This permited the direct comparison of pairs of matrices. In this study, the complementary part to a value of r = 1.0 (100%) was used as an indication of a loss of information. All analyzes were performed through the R Software, using the protest function in the vegan package [40].

## Results

**Figure 2:**
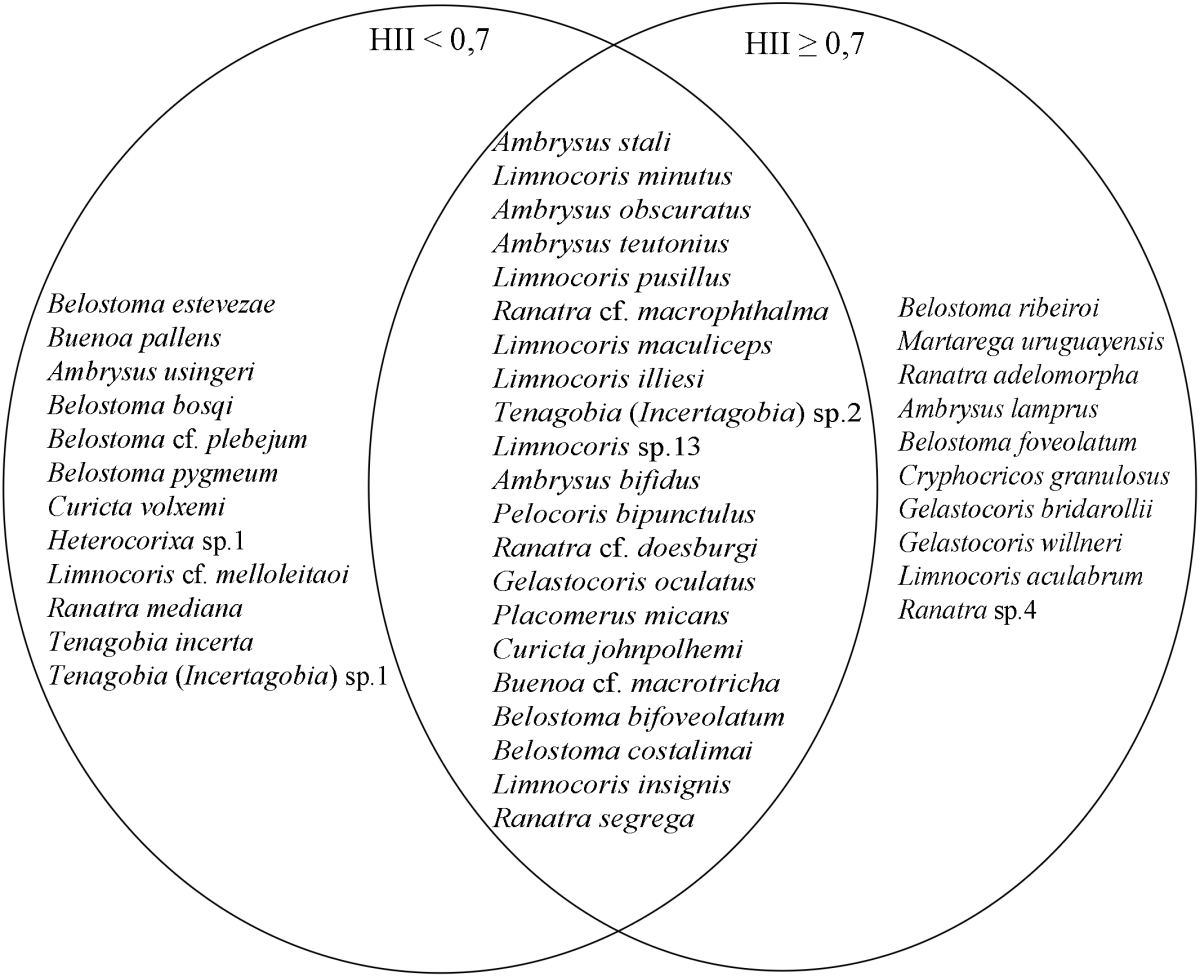
Species and morphospecies occurring in the streams surveyed in the Pindaíba River basin, Mato Grosso, Brazil.

We collected 465 Nepomorpha specimens representing six families, 13 genera, and 43 species. When the incidence of Nepomorphan at the different sites was ordered by the HII, 10 species occurred only at sites with an integrity of >0.7 and 12 only where HII was <0.7 (Figure 2). Most of these species were relatively rare (n≤4 specimens), except for *Belostoma estevezae* Ribeiro & Alecrim, 2008 (n = 17) and *B. ribeiroi* De Carlo, 1993 (n = 9).

### Taxonomic resolution – genera *versus* species

The ordination of the abundance data for genera was similar to that for species, with >80% similarity for the whole data set (r_all_ = 0.84, p<0.05) and similar values for the two types of habitat (r_alter_ = 0.92 and r_preserv_ = 0.81, p<0.05). There was thus a 16% loss of information when analyzing all sites together, 8% for altered sites only and 19% for preserved sites (Table 1).

**Table 1:** Taxonomic and numerical resolutions between data sets (abundance and incidence) for nepomorphan genera and species in Cerrado streams in the Pindaíba River basin, Mato Grosso, Brazil.

Data sets	r_all_	*p*	r_alter_	*p*	r_preserv_	*p*
Species abundance *vs.* Genus abundance	0.84	<0.001	0.92	<0.001	0.81	= 0.011
Species abundance *vs.* Species incidence	0.94	<0.001	0.95	<0.001	0.97	<0.001
Species abundance *vs.* Genus incidence	0.77	<0.001	0.91	<0.001	0.84	= 0.031
Genus abundance *vs.* genus incidence	0.74	<0.001	0.89	<0.001	0.80	= 0.012

### Numerical resolution – abundance *versus* presence/absence

The ordination patterns of the species presence/absence data were highly congruent (>94% similarity) when compared to the data on species abundance. As a consequence, the loss of information was reduced: 6, 5 and 3% (r_all_ = 0.94, r_alter_ = 0.95, and r_preserv_ = 0.97, p<0.05), respectively. On the other hand, when considering the incidence matrix for genera, despite a degree of congruence, there was an increased loss of information in comparison with the species data, i.e. 26, 11 and 20% (r_all_ = 0.74, r_alter_ = 0.89, and r_preserv_ = 0.80, p<0.05), results similar to those obtained for the comparison of the species abundance matrix with the genus presence/absence data (r_all_ = 0.77, r_alter_ = 0.91, and r_preserv_ = 0.84, p<0.05).

## Discussion

### Effects of taxonomic resolution

The results of the taxonomic resolution indicated a high degree of congruence in the ordination of matrices generated for genera and species, showing that the distribution of the Nepomorpha community was represented adequately when using the genus level, with >80% similarity. This congruence may be explained by the small number of species found in each genus, given that more than half of the genera were represented by only one or two species, and that the genera with the highest species richness were also those that were most abundant and most amply distributed. This reduced diversity is characteristic of predator taxa, such as the Plecoptera, for example. A previous study of macroinvertebrate communities [41] regarded congruence values of over 0.75 to be indicators of good levels of congruence, and in our study, which analyzed only the Nepomorpha, all the values were always similar to or above this threshold. Thus, our results indicate that it is safe to use genus-level, rather than species data, in monitoring studies, although caution is a recommended when congruence values are lower than 0.75.

This taxonomic resolution approach has been tested and applied in a number of different types of study, including the monitoring of communities, the evaluation of pollution in freshwater and seawater habitats, as well as the analysis of environmental gradients, demonstrating congruence between different taxonomic levels, even when focusing on different taxonomic groups (e.g. macrobenthos [14]; invertebrates and diatoms [42]; macroinvertebrates [43]–[45]; and phytoplankton [9], [46].

A number of recent studies analyzing different levels of taxonomic resolution in macroinvertebrates under bio-evaluation have found that higher taxonomic levels provide relevant information on environmental status [47], [26], and when species are aggregated on a higher taxonomic level, some environmental or biological information is lost [45]. By contrast, taxonomic difficulties can lead to errors of identification, which may result in major problems of interpretation [11], [48]. The balance between these opposing tendencies will depend on the purpose of the study.

In the present study, we observed that, in the Nepomorpha, there was reduced taxonomic congruence when sites were more preserved. This may be due to the fact that these sites have suffered little environmental change, and that any change would be reflected more effectively at the species level. However, the speed at which environmental changes are occurring in tropical aquatic ecosystems, and the delay in reaching the species level for data analysis, justifies the use of a genus-level taxonomic resolution. The analysis of genera rather than species should nevertheless be considered with caution, given that, while this level of analysis may be suitable for one ecosystem or region, it may not be equally effective elsewhere [41], [49]. It may also vary according to the index used, the type of analysis adopted, and the group of organisms [50], [51].

In any case, identification to the species level is not always possible, given the paucity of taxonomic information (Linnean deficit) and the lack of geographic distribution data, that is, a Wallacean deficit [10], [52], [53], as well as the inadequate number of taxonomists, identification keys, and reference collections. Moreover, the reliable morphological identification of species is often only possible for adult male and larvae in the last aquatic instar stage [48]. As a result, many of the specimens remain unidentified and are excluded from the study. According to [54], there is a payoff between the taxonomic resolution adopted and the clarity of the pattern or effect, which must be taken into account before determining the level of data resolution most appropriate for the study. Given the congruence found between the data matrices and the difficulty of identifying species reliably, our results indicate that a genus-level approach should be used in monitoring studies, considering costs and benefits (8), lower demands for time and resources, and reduced taxonomic errors compared to a species–level approach. The resources saved by choosing an optimal taxonomic (and numerical) resolution may be allocated to other purposes, such as ensuring temporal continuity and increasing spatial coverage in biomonitoring programs [9], [39], something which may increase the reliability of the results, given that communities may change depending on a wide range of environmental factors [55].

### Effects of numerical resolution

In the present study, we found a lower degree of similarity between the abundance matrices (for species or genera) and the presence/absence of genera when compared with that of species, indicating that monitoring studies based on a genus-level approach must use data on the abundance of taxa to guarantee reliability. Incidence data would nevertheless be sufficient for a species-level study. This is probably due to the fact that the community was made up of many rare species and a few dominant ones, a typical pattern in many ecosystems [56].

However, studies of aquatic macroinvertebrates, [43] have shown that data on the abundance of taxa provide the basis for more reliable interpretations than simply recording the presence/absence of taxa, although in monitoring studies, it is often difficult to measure abundance accurately. In general, these studies are based on rapid surveys, which means that reporting occurrence may be safer and more reliable. Presence/absence data also permit comparisons between different taxonomic [16] or inventories with different sampling methods. According to [57], many monitoring studies are based on presence/absence data, rather than abundance estimates, due to their vulnerability to sampling errors.

## Conclusion

The data matrix of Nepomorpha species was similar to that of the genera, and the incidence matrices of genera and species were similar to those of abundance. There was a loss of information of the order of 11–19% when the abundance matrix of genera was used instead of that of species. The congruence between the genus and species matrices indicates that, a genus-level approach is adequate for monitoring studies of nepomorphans that aim to identify environmental impacts quickly and at low cost. The potential loss of information incurred by considering only the presence/absence of taxa, supports the use of abundance data to guarantee the reliability of analyses.

Taxonomic congruence decreased at better preserved sites. This may be due to the fact that these sites presented little environmental modification and that the detection of changes would require a more refined level of taxonomic identification, that is, species rather than genus.

## Supporting Information

Appendix S1 **List of the sites surveyed in Mato Grosso state, Brazil (2005–2007/08), their respective acronyms, and geographic coordinates.** The numbers 1–4 after an acronym refer to the classification of the river, following [29]. Privately-owned areas [O = Owner of the farm; M = farm manager; Faz = Farm].
(DOCX)
