# Supplementary material for: Taxonomic and Numerical Resolutions of Nepomorpha (Insecta: Heteroptera) in Cerrado Streams
Source: PLoS One. 2014 Aug 1;9(8):e103623. doi: 10.1371/journal.pone.0103623 (PMC4118906; doi:10.1371/journal.pone.0103623)
Supplement: Appendix S1 — List of the sites surveyed in Mato Grosso state, Brazil (2005–2007/08), their respective acronyms, and geographic coordinates. The numbers 1–4 after an acronym refer to the classification of the river, following [29]. Privately-owned areas [O = Owner of the farm; M = farm manager; Faz = Farm]. (DOCX) [file pone.0103623.s001.docx]

Appendix: List of the sites of Mato Grosso State, Brazil (2005-2007/08), its respective acronyms, and geographic coordinates. The numbers 1-4 after an acronym refer to [27], classification in the river. Private areas [Owner of the farm (O); the farm manager (M); Faz - Farm].

| **Stream name** | **Order** | **Acronyms** | **Geografical coordinates** | **Farm name** | **O or M** |
| --- | --- | --- | --- | --- | --- |
| C. Cachoeirinha | 1 | SC1 | S 14º50'30", W 52º24'54" | União | O: J.Zuffo |
|  | 2 | SC2 | S 14º50'50", W 52º24'22" | União | O: J.Zuffo |
|  | 3 | SC3 | S 14º50'33", W 52º21'34" | União | O: J.Zuffo |
|  | 4 | SC4 | S 14º49'44.7'', W 52º12'56.3'' | Marca Agropecuária | O: E.Moura |
| C. Caveira | 1 | SCV1 | S 14º55.9'06'', W 52º20'29'' | Sta. Cândida | O: A.Benedine |
|  | 2 | SCV2 | S 14º59'53.4'', W 52º18'17.5'' | Brasil | M: N.Sami |
|  | 3 | SCV3 | S 14º57'28.7'', W 52º13'43.9'' | Capitão | O: V.Francisconi |
|  | 4 | SCV4 | S 14º42'47.7”, W 52º03'16.4'' | Tundavala | O: F.Ribeiro |
| C. da Mata | 1 | SM1 | S 14º29'51.7'', W 52º2'42.6'' | Carvalina | O: C.Carvalim |
|  | 2 | SM2 | S 14º59'25.2'', W 52º27'57.7'' | Carvalina | O: C.Carvalim |
|  | 3 | SM3 | S 14º59' 59'', W 52º26'29'' | Carvalina | O: C.Carvalim |
|  | 4 | SM4 | S 14º01'37'', W 52º26'29'' | Tapejara | O: A.Bressari |
| C. Papagaio | 1 | SP1 | S 15º27'01", W 52º24'30" | Pedra Branca | M: Prachá |
|  | 2 | SP2 | S 15º27'32", W 52º24'42" | Pedra Branca | M: Prachá |
|  | 3 | SP3 | S 15º28'11", W 52º24'32" | Pedra Branca | M: Prachá |
|  | 4 | SP4 | S 15º28'56", W 52º21'47" | Pedra Branca | M: Prachá |
| C. Taquaral | 1 | ST1 | S 15º41'54", W 52º20'03" | Taquaral | M: Prachá |
|  | 2 | ST2 | S 15º41'57", W 52º19'56" | Taquaral | M: Prachá |
|  | 3 | ST3 | S 15º39'35", W 52º13'52" | Sta. Catarina | M: José Maria |
|  | 4 | ST4 | S 15º38'53", W 52º12'53" | Sta. Catarina | M: José Maria |
